# A Rare Cause of Lobular Panniculitis: Munchausen's Syndrome

**DOI:** 10.1155/2012/486421

**Published:** 2012-02-27

**Authors:** Ajili Faida, Smiti Khanfir Monia, Hamzaoui Amira, Ben Ghorbel Imed, Lamloum Mounir, Houman Mohamed Habib

**Affiliations:** ^1^Department of Internal Medicine, Military Hospital of Tunis, 1008 Tunis, Tunisia; ^2^Department of Internal Medicine, La Rabta University Hospital, Tunis, Tunisia

## Abstract

We describe a case of a 40-year-old woman who presented with ecchymoses of the right leg and who was found to have lobular panniculitis in biopsy due to Munchausen's Syndrome.

## 1. Introduction

Panniculitis artefacta is the result of self-induced trauma of the skin and often manifests as polymorphic and diverse lesions. We present a case of Munchausen's Syndrome which presented as a fictitious lobular panniculitis.

## 2. Presentation

**Figure 1:**
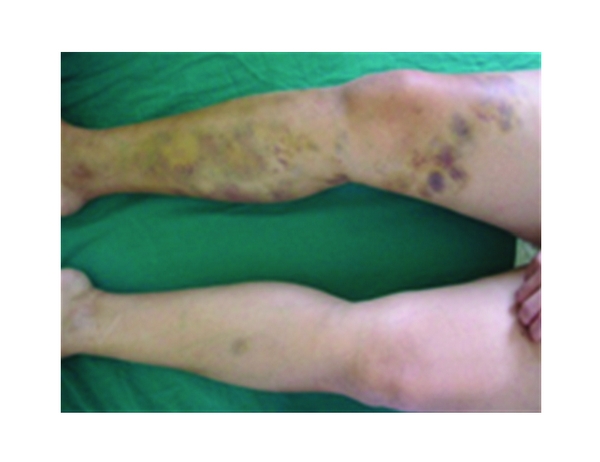
Unilateral ecchymoses.

**Figure 2:**
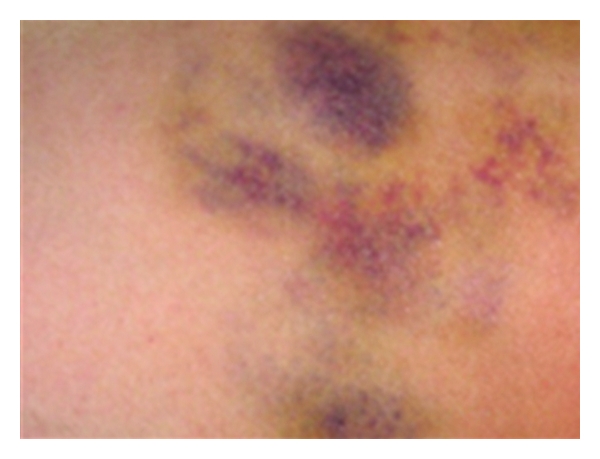
Ecchymoses of the patient's right leg.

A 40-year-old female was hospitalised in January 2006 to investigate numerous painful unilateral ecchymoses found on her right lower leg (Figures 1 and 2). The patient also complained of polyarthritis of the large joints, bi-temporal headaches, and an aphthous ulcer of the oral mucosa. She denied any history of trauma. The patient's past medical history included iron-deficiency anaemia, a duodenal ulcer, a double fracture of the right leg of unknown origin (2001) and a lumpectomy (2002). With regard to the patient's family and social history, she was living alone, unemployed, and had no children.

On physical examination, numerous painful unilateral ecchymoses and painful cutaneous/subcutaneous nodes below the surface of the ecchymoses was noted. No other abnormality were noted. 

During hospitalisation, new ecchymoses were noted, as well as a general worsening of the patient's pre-existing lesions. In addition, the patient began to complain of paraesthesia and swarming in her right lower leg. She reported a worsening of the symptoms and could no longer walk on the right leg. Initial investigations such as clotting times, immunological tests (antinuclear antibodies, anticytoplasmic neutrophil antibodies, and complement) and an echo-doppler of the lower limbs were all normal. An electromyogram was performed and showed decreased amplitude in the right lower limb suggesting peripheral neuropathy.

**Figure 3:**
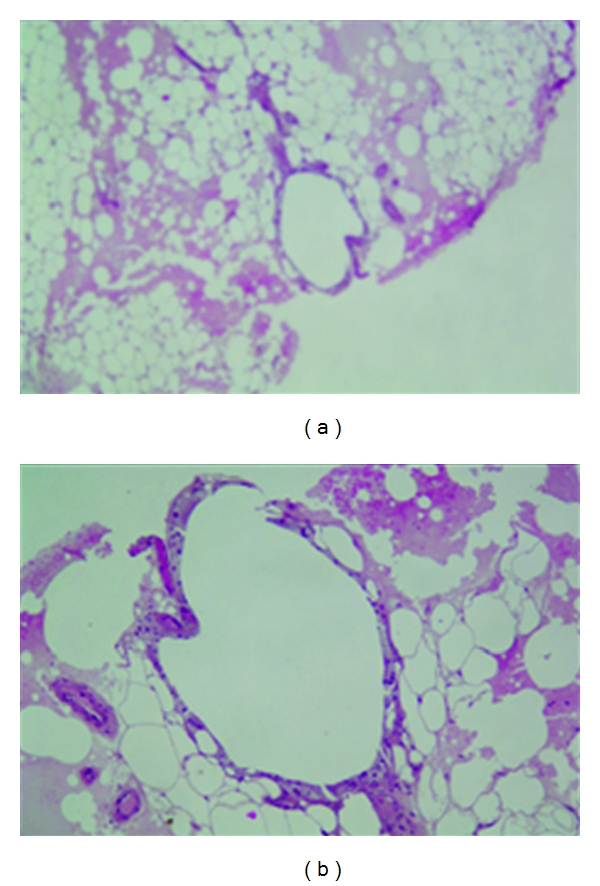
(a) (×100) and (b) (×400) coloration HE: lobular inflammatory infiltrate and adipocyte necrosis.

Histological examination of a skin biopsy revealed lymphocytic vasculitis. Histological examination of a node biopsy showed normal epidermal and dermal tissue with normal vasculature. In the hypodermis, in coloration HE, there were areas of adipocyte necrosis, lobular inflammatory infiltrate and foam cells (Figures 3(a) and 3(b)). The hypodermal septa did not appear thickened nor was there evidence of inflammatory infiltrate or vasculitic lesions. These findings show evidence of a lobular panniculitis. 

**Figure 4:**
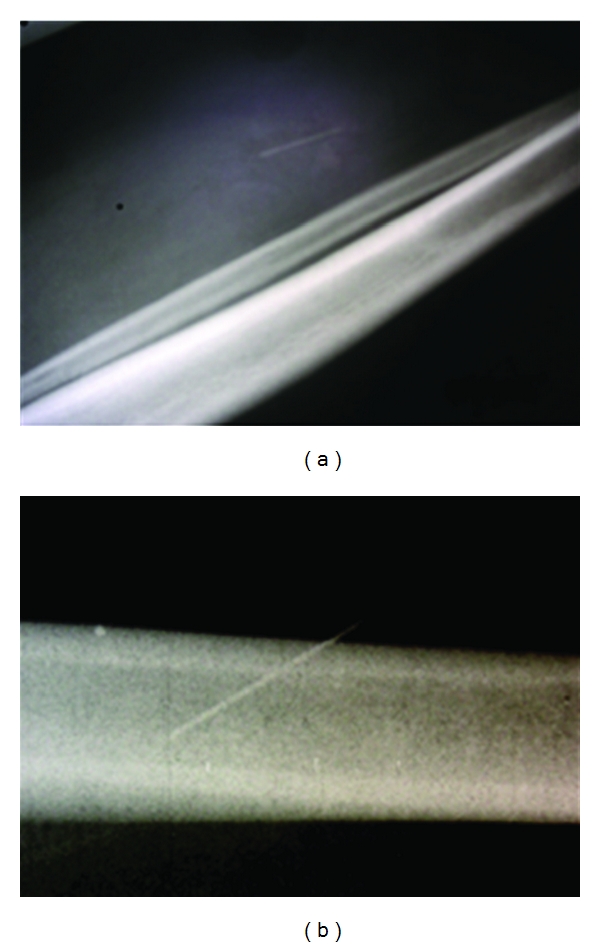
(a) and (b) Standard X-ray of the lower right leg showing presence of the sewing needle.

The patient then complained of arthritis-like symptoms in the right ankle however no signs of fever were noted, nor was there evidence of hyperleukocytosis. Several days later, the symptoms resolved spontaneously. A standard X-ray of the right leg was taken and showed the presence of a sewing needle in the patient's right calf (Figures 4(a) and 4(b)). When confronted with the question of self-inflicted lesions, the patient became aggressive and denied any knowledge of how the sewing needle got into her calf. In retrospect, another patient had reported seeing this patient put the needle into her calf. 

She then attempted to jump out of the hospital window. Hospital staff were able to stop her, and when interviewed again, she admitted to two previous suicide attempts. Following this incident, the patient refused any further medical assistance or psychiatric followup and discharged herself from hospital. It was later reported that the patient continued to consult an orthopaedic surgeon regarding her ankle.

## 3. Discussion

Factitious disorders, such as Munchausen's Syndrome, have been described as a broad spectrum of physical and/or psychological symptoms and signs, which are deliberately manufactured by the patient who has the further intention to gain the sick role [1].

In dermatology, self-induced skin diseases is an important differential diagnosis especially in the case of panniculitis or for lesions or wounds that appear artificial, geometric or found exclusively in areas that are easily accessible to the patient's hands [2]. Our diagnosis of Munchausen's Syndrome was supported by histology findings that were consistent with Panniculitis artefacta. Lobular panniculitis can either be generalised or localised. Localised panniculitis has three main differential diagnoses: trauma, cold panniculitis, or Munchausen's Syndrome. Instruments commonly used to manufacture a panniculitis include cleaning products, needles (as in our case), coins to create burns or repeated friction. The frequency of Munchausen's Syndrome is unknown, and a considerable number of cases of Munchausen's are misdiagnosed in clinical practice [2, 3].

Patients with Munchausen's Syndrome generally have a high IQ and are able to deceive physicians by producing a wide variety of signs and symptoms [4]. However, in order to exclude other possible existing pathologies, extensive clinical and laboratory examinations are often necessary. Therefore, a doctor of any specialty may face the problem of a factitious disorder and the possibility of missing the diagnosis is considerable [1, 5].

In retrospect, it is possible that in the patient diagnosed with Munchausen's Syndrome, past medical problems were also self-inflicted. Then, we concluded that our patient had Munchausen's Syndrome based on the following findings: poor historian, single female, multiple past of medical problems and hospital admissions, the well-defined, unilateral character of the ecchymoses in areas easily accessible to the hands and the peculiar evolution of these lesions, the presence of the sewing needle in her calf, and the evidence of lobular panniculitis in addition to her attempt at suicide once confronted regarding the origin of her lesions.
